# Hepatic dysfunction, hyperuricemia, and multiple renal cysts in adolescence: A case report with HNF1B deficiency and literature review 

**DOI:** 10.5414/CNCS111652

**Published:** 2026-03-25

**Authors:** Yue Yang, Ziyan Shen, Li Zhang, Wei Guo, Shaomin Gong, Jieru Cai, Jiayi Wang, Shuangxin Yuan, Xiaoqiang Ding, Xiaoyan Zhang

**Affiliations:** 1 Department of Nephrology, Zhongshan Hospital, Fudan University,; 2 Shanghai Institute of Kidney and Dialysis,; 3 Shanghai Key Laboratory of Kidney and Blood Purification,; 4 Shanghai Clinical Research Center for Kidney Diseases, and; 5 Department of Laboratory, Zhongshan Hospital, Fudan University, Shanghai, China; * These authors contributed equally to this work.

**Keywords:** 17q12 microdeletion syndrome, HNF1B deficiency, liver dysfunction, hyperuricemia, multiple renal cysts

## Abstract

Background: 17q12 microdeletion syndrome is a rare genetic disorder distinguished by diabetes, urogenital abnormalities, pancreatic hypoplasia, and neuropsychiatric developmental anomalies, with hyperuricemia being an infrequent occurrence. We present a unique case of 17q12 microdeletion, encompassing 17 protein-coding genes such as HNF1B, AATF, and DDX52 (3A:0), marking the first documented instance globally. Case report: An 18-year-old female patient was hospitalized due to persistent right upper abdominal pain for more than a month. She exhibited a normal body mass index and no familial medical history. Biochemical analysis indicated liver impairment, hyperlipidemia, hypomagnesemia, hyperuricemia, and glucose intolerance. Renal ultrasound displayed numerous renal cysts, while a liver biopsy confirmed the presence of non-alcoholic fatty liver disease. Given the early onset of metabolic syndrome, whole-exome sequencing (WES) was conducted on the patient and her parents. Results: By WES and copy number variation verification, the patient was identified as having a de novo heterozygous deletion of one copy in the 17q12 region (34807034-36285028), encompassing 17 protein-coding genes including HNF1B, AATF, and DDX52 (3A:0). Tubulointerstitial lesions are the predominant feature of HNF1B-related renal disease, with hyperuricemia showing limited predictive value despite its common occurrence. Hypomagnesemia is a significant clinical indicator in HNF1B mutation-related conditions. Conclusion: Hyperuricemia, hypomagnesemia, and multiple renal cysts can manifest as renal symptoms of the microdeletion in the 17q12 region, with the spectrum of extra-renal manifestations continually expanding. For young patients exhibiting the above metabolic issues, WES is recommended for precise diagnosis.

## Introduction 

**Figure 1:**
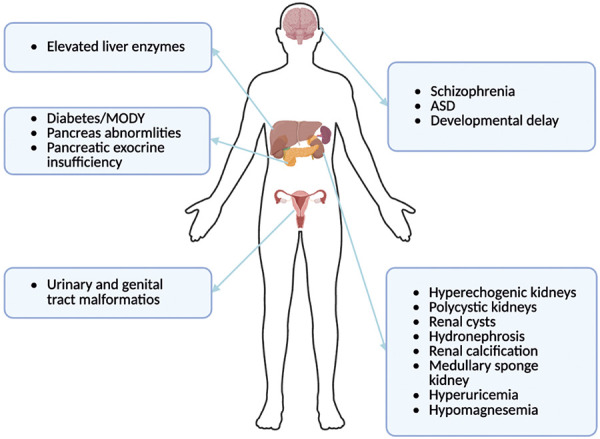
Spectrum of renal and extra-renal phenotypes in HNF1B-related disease. MODY = maturity-onset diabetes of the young; ASD = autism spectrum disorder.

Hepatic nuclear factor 1b (HNF1B), a transcription factor initially identified in human liver cells [1], plays a crucial role in tissue development and organ function maintenance. HNF1B is expressed in various tissues and organs, including the liver, kidney, lung, pancreas, intestine, and reproductive systems. In the metanephros and ureter, which are the most affected organs, this gene is strongly and specifically expressed in the epithelium, while the glomeruli are not involved [2]. The first documented heterozygous mutations of HNF1B were associated with maturity-onset diabetes of the young type 5 (MODY5) [3]. Conditions arising from HNF1B defects encompass a spectrum of renal and extra-renal manifestations, collectively known as the 17q12 microdeletion syndrome [4]. The predominant phenotypes include renal cysts and type 2 diabetes, referred to as renal cysts and diabetes (RCAD) syndrome. Extra-renal manifestations comprise pancreatic hypoplasia, hepatic dysfunction, electrolyte imbalances, and reproductive system abnormalities (Figure 1). 

HNF1B defects are inherited in an autosomal dominant manner, with newly identified pathogenic variants contributing to 50% of cases [5]. Among the various mutation types reported, whole gene deletions account for over 50%, with a majority being de novo mutations [6]. The complexity of genotypes and phenotypes presents a challenge to prompt and accurate clinical diagnosis. 

We present a case of an 18-year-old female with a de novo heterozygous deletion of HNF1B. This individual exhibited a copy number variation (CNV) in the region 17q12 (Seq[GRCh37]del(17)(q12)NC_000017.10:g.34807034-36285028) of chromosome 17, which included a total of 17 protein-coding genes such as HNF1B, apoptosis antagonizing transcription factor (AATF), and DEAD-box helicase 52 (DDX52) (3A:0). To our knowledge, this variant has not been observed in the general population and is not currently documented in any databases, making this case potentially the first reported worldwide. 

## Case report 

### Clinical manifestations 

An 18-year-old female came to Zhongshan Hospital, Fudan University on October, 2023, with the chief complaint of right upper abdominal pain for over a month. Her personal history was negative, no history of smoking or alcohol. She was the only child of her parents, with no siblings. Normal menstruation cycles. Physical examination: height 168 cm, weight 60 kg, BMI 21.3 kg/m^2^. No special facial features. 

### Laboratory tests 

Laboratory tests showed a moderate elevated transaminase, hyperuricemia, and hyperlipidemia (Table 1). Oral glucose tolerance tests revealed impaired glucose intolerance with 2-hour postprandial blood glucose 7.9 mmol/L [7, 8], and homeostasis model assessment of insulin resistance (HOMA-IR) index was 2.288 [9], which met the criteria of insulin resistance. Hepatitis A, B, C, and E virus were all negative. Thyroid function and parathyroid hormone levels were within normal range. 

Since the patient showed persistent hypomagnesemia with serum magnesium 0.54 mmol/L and 0.57 mmol/L without significant clinical symptoms, 24-hour urine analysis was performed (Table 1). Excessive renal excretion of magnesium was indicated by a 24-hour urinary magnesium excretion of 2.39 mmol/h (< 2.5 mmol/24h), and low renal excretion of uric acid was indicated by a fractional excretion of uric acid (FE_UA_) of 3.4% (< 5.5%) and urinary uric acid excretion (UE_UA_) of 1,430.5 (< 3,570) μmol/day/1.73m^2^. Therefore, hypomagnesemia and hyperuricemia (decreased excretion) were diagnosed. 

### Imaging examination and liver biopsy 

**Figure 2:**
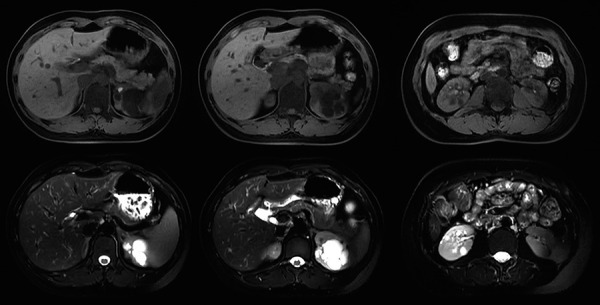
Bilateral renal cysts revealed by magnetic resonance cholangiopancreatography. MRCP reported multiple cystic lesions in the renal parenchyma without significant enhancement in contrast imaging, making the contour of bilateral kidneys irregular (Above: T1WI; Below: T2WI).

Magnetic resonance cholangiopancreatography (MRCP) examination of upper abdomen showed normal signal of liver, a slightly enlarged spleen, and chronic cholecystitis. Bilateral kidneys were also scanned, reporting multiple cystic lesions in the renal parenchyma without significant enhancement in contrast imaging, making the contour of bilateral kidneys irregular (Figure 2). The patient was then admitted to the Department of Gastroenterology for a liver biopsy. Liver biopsy revealed hydropic degeneration and focal (2%) fatty degeneration in hepatocytes, and she was diagnosed as non-alcoholic fatty liver disease (NAFLD). 

Based on hyperuricemia and bilateral multiple renal cysts, nephrology consultation was requested. Renal ultrasound was performed, and bilateral kidneys were slightly enlarged (left: 111 × 44 × 45 mm; right: 107 × 46 × 44 mm), with multiple echo-free spaces (left: 25 × 23 mm max.; right: 11 × 9 mm max.). No renal pelvis separation was observed, but multiple strong echogenic masses were seen in the right renal pelvis, indicating right renal calculi. Gynecology ultrasound reported no reproductive system abnormalities. 

### Whole-exome sequencing and functional validation 

Considering the co-existence of multiple renal cysts, impaired glucose intolerance and insulin resistance, hyperlipidemia, and NAFLD at the age of 18, we recommended the patient to perform whole exome sequencing (WES) to exclude genetic diseases. WES discovered a CNV in the region 17q12 (Seq[GRCh37]del(17)(q12)NC_000017.10:g.34807034-36285028) of chromosome 17, which included a total of 17 protein-coding genes such as HNF1B, AATF, and DDX52 (3A:0). The deleted region completely overlapped with the well-defined HI gene HNF1B (2A:1). There were no records of this variant in the population database DGV (Database of Genomic Variants). No study of this CNV was reported in relevant databases such as Clinvar and Decipher. Considering the patient’s clinical symptoms, related disease characteristics, and genetic testing results and according to the ACMG/AMP and ClinGen guidelines for interpreting variations, this CNV detected in the patient was classified as a pathogenic variant. 

**Figure 3:**
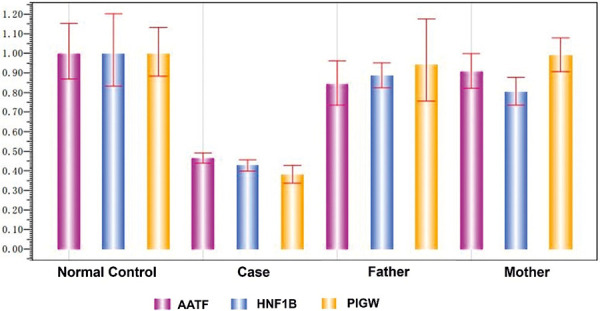
Copy number variation verification proved a de novo heterozygous deletion in the chr17:34807034-36285028. The copy number variation verification proved a heterozygous deletion in the chr17:34807034-36285028 [GRCh 37/hg19] region in the case, whereas her parents had normal copy numbers in this region.

Variant validation of the CNV was performed on proband and family unaffected individuals by real-time quantitative PCR (qPCR). Proband was heterozygous with chr17:34807034-36285028 deletion, and both parents had a normal copy number in this region. This result indicated that this CNV was a de novo deletion for proband (Figure 3). 

### Treatment and follow-up 

To alleviate the liver dysfunction and hyperlipidemia, fenofibrate, compound glycyrrhizin, and ursodeoxycholic acid were prescribed. To reduce serum uric acid, febuxostat was prescribed. Symptoms were relieved after medication. Liver function, hyperlipidemia, and hyperuricemia were all relieved. Over this period, clinical symptoms improved, and laboratory findings showed normalization of liver function tests, a significant decrease in lipid levels, and a reduction in serum uric acid to within the normal range. No adverse effects were observed during treatment, and the patient remained stable at the most recent follow-up. 

## Discussion 

There is a significant heterogeneity between the genotype and phenotype in HNF1B-related disease [6]. Renal involvement is the most prominent and characteristic finding, which mainly include renal malformations and tubular transport abnormalities. Among these features, the most frequent presentation consists of bilateral hyperechogenic kidneys with or without cortical cysts. Other renal phenotypes include bilateral hydronephrosis, renal calcification, and nephrogenic diabetes insipidus. Medullary sponge kidney (MSK) was reported in an HNF1B whole-gene deletion case [10]. Most common extrarenal presentations in RCAD syndrome included: maturity-onset diabetes of the young type 5 (MODY5), genital abnormalities [11], hyperparathyroidism, liver dysfunction as well as structural and exocrine abnormalities of the pancreas [12]. Also, there is an increased risk of neuropsychiatric disorders [13]. 

In our case, our patient presented with liver dysfunction, NAFLD, multiple bilateral renal cysts and renal calculi, hyperuricemia, hypomagnesemia, hyperlipidemia, impaired glucose intolerance and insulin resistance at adolescence. Due to the uncertainty of clinical phenotype, whole-exome sequencing was performed, and definite diagnosis was confirmed. 

Based on a retrospective study, ~ 40% of individuals with HNF1B defects may display signs of liver dysfunction, with a higher prevalence observed in those with MODY5 phenotype [14]. Liver dysfunctions associated with HNF1B are typically identified by increased liver enzymes and histological evidence of hepatic steatosis, mirroring the characteristics observed in our current case. Neonatal cholestasis has also been reported [15]. 

As the most common manifestation, the mechanism underlying polycystic kidney remains unclear. Frequently mutated genes in polycystic kidney diseases comprise PKHD1, PKHD2, UMOD, and GLIS27. Mutations in HNF1B could potentially lead to the downregulation of these genes associated with cystic diseases, thereby fostering cyst formation. Additionally, the aberrant expression of Ksp-cadherin might play a role in the developmental anomalies observed in the kidney and genitourinary tract of individuals with HNF1B abnormalities [17]. 

In comparison to normal control subjects and individuals with type-2 diabetes, HNF1B defect subjects exhibited significantly elevated serum urate levels [18, 19]. The association between elevated serum uric acid and HNF1B defects is established, although the precise underlying mechanism remains elusive. It is well-established that serum uric acid is primarily excreted through the kidneys, and an elevated serum uric acid is indicative of compromised renal function [20]. Notably, the characteristics of HNF1B-related hyperuricemia appear disproportionate to the extent of renal dysfunction, with a low fractional excretion of uric acid [18]. One hypothesis is that hyperuricemia may be linked to increased reabsorption in the proximal tubule secondary to salt wasting in the urine [21]. 

The prevailing belief is that damage to the functional and structural integrity of Henle’s loop serves as a pivotal pathogenic factor, leading to impaired urine concentration and subsequent hyperuricemia. In our study, hyperuricemia in HNF1B-related disease was classified as “decreased excretion” based on FE_UA_ and UE_UA_ calculations. However, despite being a prominent feature of HNF1B-related disease, hyperuricemia does not serve as a reliable marker for the disease. A retrospective analysis in children revealed a low prevalence of hyperuricemia in HNF1B-related disease, and the inclusion of hyperuricemia in a multivariate logistic regression model did not enhance its predictive accuracy [19]. Furthermore, FE_UA_ was found to be an inadequate indicator in this context. 

Renal regulation plays a crucial role in maintaining magnesium balance within the body, while hypomagnesemia usually is an underestimated biochemical indicator of renal dysfunction. Unlike other electrolytes, magnesium reabsorption primarily occurs in the thick ascending limb of the loop of Henle, with only a small percentage (3 – 6%) ultimately being excreted by the kidneys. In certain instances, hypomagnesemia may present as an initial symptom. Intriguingly, individuals with HNF1B deletions appear to have a higher propensity for developing hypomagnesemia compared to those with intragenic mutations [22]. The significance of hypomagnesemia as a predictive marker for HNF1B mutations may have been underestimated in previous assessments [19]. Renal cysts combined with hypomagnesemia may be a strong indicator of HNF1B defect. 

The mechanism by which HNF1B influences and regulates the reabsorption, transport, and excretion of magnesium is speculated to be linked to the FXYD2 gene. FXYD2 encodes the regulatory subunit of Na+- K+-ATPase, a key player in modulating the reabsorption of sodium ions in the renal tubules. HNF1B may impact epithelial ion transport by regulating FXYD2 expression [23]. A newly identified target of HNF1B-induced transcription is Kir5.1, offering fresh insights into abnormal electrolyte wasting [24]. Hypercalciuria often coexists with hypomagnesemia and hypokalemia, but the exact pathophysiological mechanism remains unclear. It is yet to be determined whether the inappropriate depletion is a result of HNF1B mutations, prolonged hypomagnesemia, or a combination of both factors [14]. There may be a potential correlation with the calcium-sensing receptor in the thick ascending limb of the kidney, further highlighting the intricate interplay of these regulatory mechanisms [25]. 

In our case, based on the thorough serum electrolyte and urine tests, the patient was diagnosed with hypomagnesemia and inappropriate renal magnesium excretion, after ruling out the use of diuretics, hypercalcemia, diabetes, hypoparathyroidism, hyperthyroidism, and other potential contributing factors. 

MODY5, which arises from mutations in HNF1B, represents a monogenic form of diabetes characterized by an onset age of under 25 and an autosomal dominant inheritance pattern. Despite accounting for at least 1% of diabetes mellitus cases, MODY5 cases are frequently misclassified as either type 1 or type 2 diabetes [26]. Intriguingly, individuals with HNF1B-related diabetes may exhibit a reduced susceptibility to microvascular complications [14]. The underlying pathophysiological mechanisms of MODY5 remain inadequately elucidated, possibly due to the challenges in developing appropriate animal models. Nevertheless, previous investigations have indicated that an early deficiency in HNF1B leads to a diminished pool of pancreatic progenitor cells [27]. In our patient’s case, impaired glucose tolerance and insulin resistance have already manifested, underscoring the importance of vigilant monitoring of blood glucose levels and the prompt initiation of appropriate treatment during subsequent follow-up assessments. 

In summary, we have documented a case of hepatic dysfunction accompanied by renal cysts and hyperuricemia during adolescence, featuring a novel deletion mutation site at chr17:34807034-36285028 [GRCh 37/hg19]. This discovery serves to expand the genetic mutation spectrum associated with the 17q12 deletion syndrome. Our thorough examination of the patient allowed for a comprehensive assessment of the disease’s manifestations. For specialists in nephrology, urology, endocrinology, and gastroenterology, the presence of two or more manifestations such as polycystic kidneys, hyperuricemia, hypomagnesemia, hyperlipidemia, impaired glucose tolerance, and liver dysfunction in young patients should prompt consideration of a potential genetic disorder. 

## Informed consent statement 

Informed consent has been obtained from the patient to publish this manuscript. The study was conducted ethically in accordance with the World Medical Association Declaration of Helsinki and approved by the Ethical Committee of Zhongshan Hospital (CR2024-011). 

## Authors’ contributions 

XYZ and ZYS contributed to the conception and design of the work. SMG, JRC, JYW, SXY, LZ, and WG contributed to the acquisition, analysis, and interpretation of data for the work. YY drafted the manuscript. XYZ, ZYS, and LZ critically revised the manuscript. ZYS and XQD provided the funding. All authors gave final approval and agree to be accountable for all aspects of work, ensuring integrity and accuracy. 

## Funding 

The study was funded by the National Natural Science Foundation of China (82002018), Shanghai Sailing Program (20YF1406000), Shanghai Clinical Research Center for Kidney Disease (22MC1940100), and Shanghai Federation of Nephrology Project supported by Shanghai ShenKang Hospital Development Center (SHDC22022309). 

## Conflict of interest 

All authors declare that there is no conflict of interest. 

**Table 1.** Laboratory findings.

Urine analysis		Blood chemistry (2)	
pH	5.50	Ceruloplasmin	215 mg/L
Urine-specific gravity	1.003	LDH	108 (109 – 245) U/L
Protein	Negative (0.07 g/d)	cholinesterase	8,420 (5,000 – 12,000) U/L
RBC	negative	Pre-albumin	228 (180 – 350) mg/L
WBC	0 – 2/HP	Total cholesterol	6.43 (0 ~ 5.2) mmol/L
Casts	Not detected	Triglyceride	3.00 (0 ~ 1.7) mmol/L
Bence Jones protein	Not detected	HDL cholesterol	1.35 (0 ~ 4.1) mmol/L
Blood count		LDL cholesterol	3.72 (0 ~ 3.4) mmol/L
WBC	6.88 (3.50 – 9.50) ×10^9^/L	Fasting blood glucose	5.2 (3.9 – 5.6) mmol/L
RBC	4.05 (3.80 – 5.10) ×10^12^/L	30 min blood glucose	9.7
Hb	115 (115 – 150) g/L	1 h blood glucose	11.5
Plt	232 (125 – 350) ×10^9^/L	2 h blood glucose	7.9
Blood chemistry (1)		3 h blood glucose	7.1
Total protein	65 (65 – 85) g/L	Fasting insulin	9.9(2.6 – 24.9) mU/mL
Albumin	43 (35 – 55) g/L	30 min insulin	88.4
AST	101 (13 – 35) U/L	1 h insulin	146.0
ALT	165 (7 – 40) U/L	2 h insulin	198.0
ALP	224 (35 – 100) U/L	3 h insulin	142.0
γ-GT	340 (7 – 45) U/L	Urine chemistry	
Na	146 (137 – 147) mmol/L	Na	62 (130 – 260) mmol/24h
K	4.0 (3.5 – 5.3) mmol/L	K	38.9 (25 – 100)
Cl	107 (99 – 110) mmol/L	Cl	76 (170 – 250)
Ca	2.33 (2.15 – 2.55) mmol/L	Ca	0.42 (2.5 – 8.0)
P	1.38 (0.90 – 1.34) mmol/L	P	16.6 (12.9 – 42.0)
Mg	0.54 (0.67 – 1.04) mmol/L	Mg	2.39 (2.5 – 8.0)
BUN	4.2 (2.9 – 8.2) mmol/L	Urea	240.3 (430 – 720)
Creatinine	59 (44 – 115) μmol/L	Creatinine	6,340 (6,300 – 13,400) μmol/L
Uric acid	633 (155 – 357) μmol/L	Uric acid	2,346 (1,200 – 5,900)
Estimated GFR (CKD-EPI formula)	> 120 mL/min/1.73m^2^	Glucose	< 0.11 mmol/24h
		Fractional excretion	
		FE_UA_	3.4%
		FE_Mg_	5.88%

Reference values are given in parentheses. RBC = red blood cell; WBC = white blood cell; HP = high power; Hb = hemoglobin; Plt = platelets; BUN = blood urea nitrogen; GFR = glomerular filtration rate; AST = aspartate aminotransferase; ALT = alanine aminotransferase; LDH = lactate dehydrogenase; ALP = alkaline phosphatase; γ-GT = γ-glutamyltransferase; HDL = high-density lipoprotein; LDL = low-density lipoprotein; FE_UA_ = fractional excretion of uric acid, FE_UA_(%) = (urine uric acid × serum creatinine) / (serum uric acid × urine creatinine) × 100%; FE_Mg_, = fractional excretion of Mg, FE_Mg_(%) = (urine Mg × serum creatinine) / (serum Mg × urine creatinine) × 100%. (1) = routine biochemistry; (2) = metabolic parameters.
